# *DDI-CPI*, a server that predicts drug–drug interactions through implementing the chemical–protein interactome

**DOI:** 10.1093/nar/gku433

**Published:** 2014-05-29

**Authors:** Heng Luo, Ping Zhang, Hui Huang, Jialiang Huang, Emily Kao, Leming Shi, Lin He, Lun Yang

**Affiliations:** 1 Bio-X Institutes, Shanghai Jiao Tong University, Shanghai 200030, China; 2 University of Arkansas at Little Rock/University of Arkansas for Medical Sciences, Little Rock, AR 72204, USA; 3 Healthcare Analytics Research Group, IBM T.J. Watson Research Center, Yorktown Heights, NY 10598, USA; 4 Department of Biostatistics and Computational Biology, Dana-Farber Cancer Institute and Harvard School of Public Heath, Boston, MA 02215, USA; 5 Department of Bioengineering, University of California at Berkeley, Berkeley, CA 94720, USA; 6 School of Pharmacy, Fudan University, Shanghai 201203, China

## Abstract

Drug–drug interactions (DDIs) may cause serious side-effects that draw great attention from both academia and industry. Since some DDIs are mediated by unexpected drug–human protein interactions, it is reasonable to analyze the chemical–protein interactome (CPI) profiles of the drugs to predict their DDIs. Here we introduce the *DDI-CPI* server, which can make real-time DDI predictions based only on molecular structure. When the user submits a molecule, the server will dock user's molecule across 611 human proteins, generating a CPI profile that can be used as a feature vector for the pre-constructed prediction model. It can suggest potential DDIs between the user's molecule and our library of 2515 drug molecules. In cross-validation and independent validation, the server achieved an AUC greater than 0.85. Additionally, by investigating the CPI profiles of predicted DDI, users can explore the PK/PD proteins that might be involved in a particular DDI. A 3D visualization of the drug-protein interaction will be provided as well. The *DDI-CPI* is freely accessible at http://cpi.bio-x.cn/ddi/.

## INTRODUCTION

A recent study indicates that drugs are commonly co-prescribed, and nearly one out of 25 individuals are at risk of a major adverse reaction caused by drug–drug interactions (DDIs), especially in older patients (1). In addition to severe adverse reactions, DDIs may result in early termination of drug developments as well as withdrawal of marketed drugs (2,3). Predicting and discovering DDIs will not only prevent life-threatening consequences in clinical practice, but also prompt safe drug co-prescriptions for better treatments (4,5).

DDIs can be classified into three categories: pharmaceutical, pharmacokinetic (PK) and pharmacodynamic (PD) (6). Pharmaceutical interactions are usually caused by physical or chemical incompatibility among the co-prescribed drugs. PK interactions refer to the perturbations on the absorption, distribution, metabolism or excretion of one another, which are usually mediated by PK proteins (7). PD interactions create antagonistic or synergistic pharmacologic effect of two drugs (8) which may involve unexpected bindings of the drug molecules with the PD proteins, such as target or off-target proteins (9,10). There are published computational methodologies predicting DDIs (4,8,11–17); however, as far as we know, no research has published the prediction model based only on drug structure without requiring additional pharmacological or biological background information of the predicted drug. Also, there are currently no freely available servers for real-time DDI predictions.

As many of the DDIs are mediated by unexpected drug-protein interactions, it is reasonable to utilize such interactome information to make DDI predictions. Therefore, we introduce *DDI-CPI*, a server predicting drug–drug interactions via chemical–protein interactome (CPI). The CPI is a methodology that utilizes *in silico* simulations to mimic the theoretical interaction profile (docking results) of a small molecule across human proteome (9,18–22). Since CPI has been applied in predicting drug's pharmacological effects such as adverse drug reaction (23–25) and drug repositioning potential (26), we implement the CPI methodology in the DDI-CPI server. The server collects high-quality structures of ligand-bindable human proteins from third-party human curated databases, including PK and PD proteins. When processing user's submitted molecule, the server will calculate the theoretical free energy of bindings for it across the entire panel of human PK/PD proteins, generating a vector of interaction strengths for the prediction model. It can alert the high risk DDIs among user's molecule against 2000+ U.S. Food and Drug Administration (FDA) approved drugs, guiding the safe drug co-prescription.

Compared to other DDI prediction methods, the server has the following distinctions: (i) It predicts both PK and PD mediated DDIs; (ii) rather than using sophisticated information such as pathways or networks, the biological rationale of the prediction model is simple in explanation, such as which PK/PD proteins may be involved in this DDI; (iii) The prediction model used in our server achieves high accuracies in both cross-validation and independent validation.

## METHODS

### Preparation of the library drugs and targets

We collected 2515 library drug molecules (85% are FDA approved drugs) and annotations with different ionization states from DrugBank (27) and STITCH (28), and then prepared their 3D structures via Corina online and Vega ZZ (29). The list of drugs is attached in Supplementary Table S1.

We also collected 611 high quality ligand-bindable PDB structures, including 239 human PK proteins and 372 PD proteins. The PK proteins were Protein Data Bank (PDB) (30) structures from a published paper with all available drug metabolite enzymes (31). The PD proteins were distinct human proteins prepared from the PDBBind database (32), which contains curated crystal structures with binding pocket information. All the proteins we selected were based on the following criteria: (i) all proteins have X-ray crystal structures, (ii) all structures have better resolution than 3.4 Å (89% of the protein ended up with better resolution than 2.5 Å) and (iii) binding pockets were identified around the embedded ligands in the crystal structure (25,26). Subsequently, we extracted function annotations for those proteins from UniProtKB (33). The list of proteins is included in Supplementary Table S2.

### Preparation of the CPI

AutoDock Vina is a molecular docking program that has improved speed and accuracy in comparison to AutoDock and DOCK (34,35). The docking of all 2515 library drug molecules across 611 targetable human protein pockets were constructed using AutoDock Vina (34) with the random seed set to 10 000 and other default parameters. We implemented rigid docking rather than flexible docking to ensure a reasonable calculation speed. The minimal docking scores and corresponding docked structures were chosen as the representative docking results to be displayed later.

### The training set for DDI prediction model

**Figure 1.**
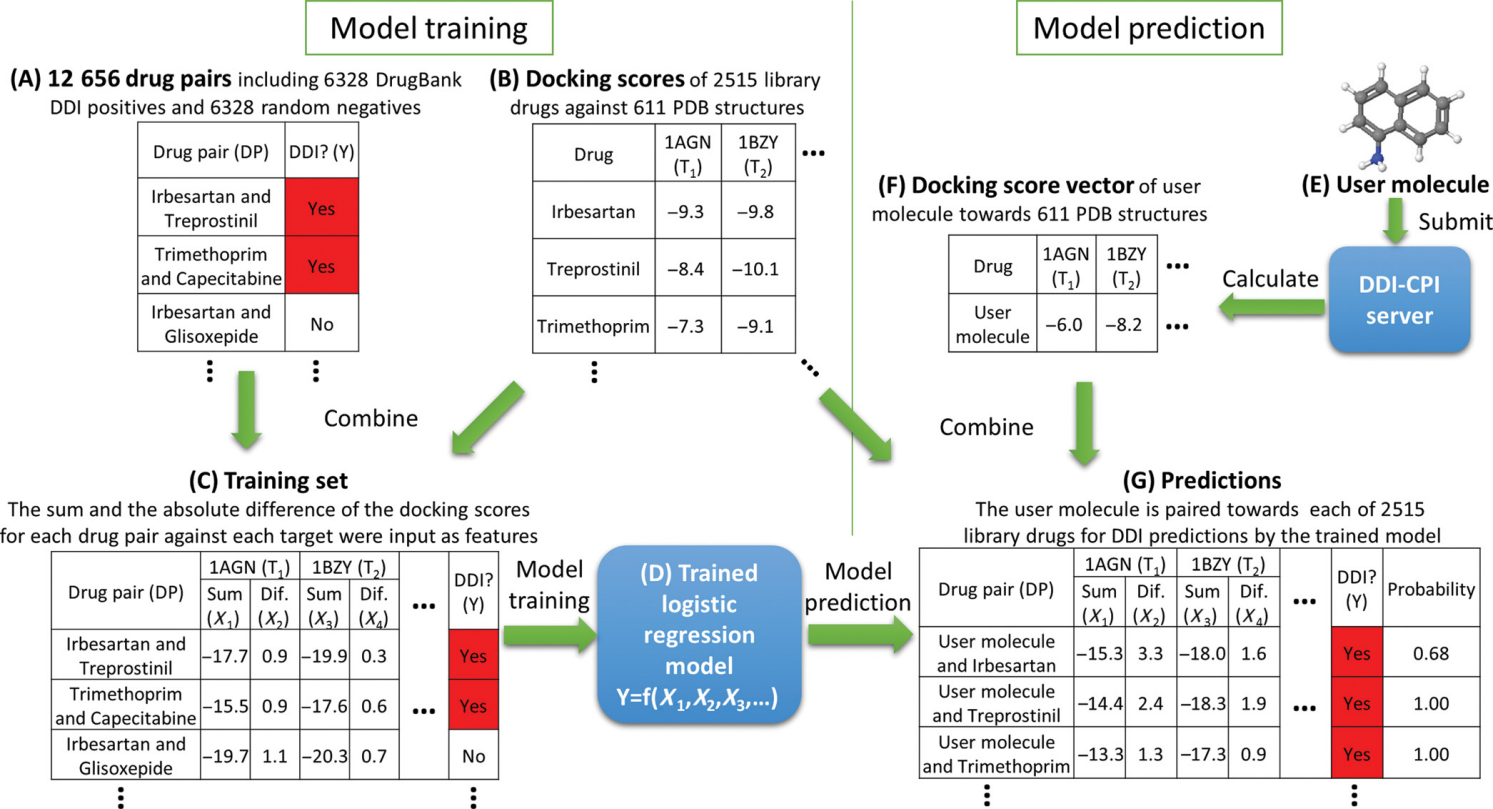
The server workflow showcasing model training and prediction. (**A**) The 12 656 drug pairs including 6328 DrugBank DDI positives and 6328 randomly generated negatives were prepared. (**B**) CPI profiles of 2515 library drug molecules across 611 PDB structures were generated using AutoDock Vina. (**C**) For each drug pair ***DP_i_*** toward each PDB target ***T_j_***, the sum and absolute difference of their docking scores were calculated and used as features. (**D**) A logistic regression model was trained based on this training set. (**E**) When the user submits a molecule, (**F**) the server calculates the CPI profile and generates the feature vector. (**G**) The user molecule is then paired with each of the 2515 drug molecules in library to form 2515 new drug pairs. 2515 feature vectors containing the sum and absolute difference of the docking scores for each drug pair were generated and sent to the trained model to make predictions.

We used DrugBank DDIs (27) and obtained 6328 drug pairs with complete CPI annotations, which was used as the positive set. According to recent DDI prediction methods (8,11), we randomly generated 6328 drug pairs that did not appear in the positive set as the negative set (Figure 1A). The DrugBank IDs of the drug pairs involved in the positive and negative sets are attached in Supplementary Table S3.

### Model training and validation

Docking scores for each drug in the training set were generated against the 611 library targets (Figure 1B). For each two drugs in drug pair *DP_i_* against target *T_j_*, we calculated the sum *S*(*DP_i_, T_j_*) and absolute value of the difference *AD*(*DP_i_, T_j_*) of their docking scores as features. Since we have 611 library targets, we could generate 1222 features for each drug pair *DP_i_*. In this way, the training set was converted to a matrix containing 12 656 drug pairs as rows and 1222 features as columns with a final column as a dependent variable (Figure 1C). A logistic regression model was trained based on this matrix for server-side predictions (Figure 1D).

To validate our method, we randomly held 50% of the original training data as an independent validation dataset. For the rest of them, we applied logistic regression using a 10-fold cross-validation to evaluate their performance. The model was set up with L2-regularization which gives an increasing penalty as model complexity increases to prevent overfitting. We repeated the cross-validation experiment 100 times to get a mean and a standard deviation of the area under receiver operating characteristic curve (AUROC) and the area under precision-recall curve (AUPR). We calculated the accuracy, precision, sensitivity and specificity measures based on a prediction threshold when the maximum *F*-score (harmonic mean of precision and recall) was achieved. Then we evaluated this model on the independent validation data. To obtain the accuracy, precision, sensitivity and specificity measures for the independent validation set, we used the average thresholds selected in the cross-validation experiments. Since this independent dataset was not included anywhere in the training, we used it as a gold standard to compare with other published prediction models.

## INPUT AND OUTPUT

Users are required to submit a molecular file with specific formats such as mol, mol2, sdf, pdb and SMILES (Figure 1E). We utilize free tools including OpenBabel (36) and Autodock Tools (37) to convert the file into PDBQT format with Gasteiger charges. An example drug molecule is provided for a quick test. When a user molecule is submitted, the docking scores of this drug toward all targets in the database is calculated via AutoDock Vina (34) with default number of poses (eight or more). This process is similar to the inverse- or reverse-docking approach (21,25,38). Here, only the lowest energy scores with the corresponding poses were selected (Figure 1F) to build the CPI profiles, which were fed to the server-side classification model to predict the DDIs (see Figure 1G for detail). The process time ranges from minutes up to several hours, and an email will be sent to the user upon completion of the task. Users can also track the real-time calculation progress online.

The user will be able to view the following outputs:

- DDI probabilities of user's molecule with 2515 drug molecules in library.
- PK/PD proteins that may be involved in the DDI. The server can visualize the 3D conformation of each drug-protein interactions via Jmol (http://www.jmol.org), with amino acid residues around 6.4 Å of the molecule highlighted.

## RESULTS

### Model evaluation

The model obtained an AUROC of 0.861 ± 0.001 and AUPR of 0.860 ± 0.001 in the 10-fold cross-validation (accuracy: 0.804 ± 0.002, precision: 0.742 ± 0.010, sensitivity: 0.847 ± 0.013, specificity: 0.772 ± 0.012, *R*^2^: 0.386 ± 0.002).

**Figure 2.**
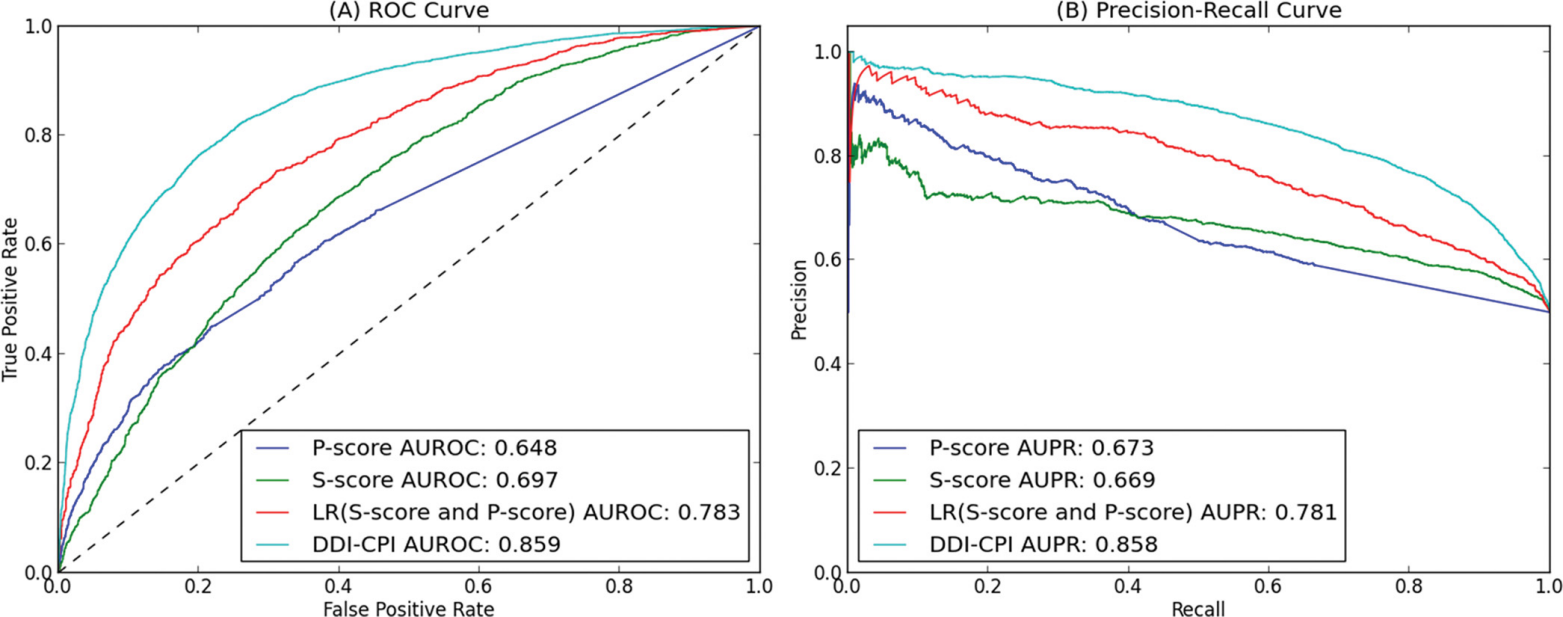
(**A**) The ROC curve comparison for different DDI prediction methods on the independent validation data. (**B**) The precision-recall curve comparison for different DDI prediction methods on the independent validation data.

Based on the independent validation data, we compared four prediction methods: (i) *P*-score that uses side-effect similarities to measure the connection between two drugs (39). (ii) *S*-score that measures the strength of network connections between drug targets to predict DDIs (8). (iii) LR (*S*-score and *P*-score) that integrates *P*-score and *S*-score by a Bayesian probabilistic model and achieves superior performance than previous prediction methods (8). (iv) DDI-CPI proposed in this paper that analyzes CPI profile to predict DDIs. The comparisons of receiver operating characteristic (ROC) curves and precision-recall curves are shown in Figure 2 and all evaluation measurements are summarized in Table 1. We could see that the CPI-based method outperformed previously-developed computational methods on different parameters.

**Table 1.** Performance comparison for different DDI prediction methods on the independent validation data

	Accuracy	Precision	Sensitivity	Specificity	AUROC	AUPR	*R*^2^
*P*-score	0.677	0.590	0.667	0.683	0.648	0.673	0.074
*S*-score	0.715	0.578	0.898	0.604	0.697	0.669	0.057
LR	0.744	0.646	0.824	0.689	0.783	0.781	0.132
DDI-CPI	0.805	0.752	0.833	0.784	0.859	0.858	0.383

### Case study: DDI prediction for sertraline

**Figure 3.**
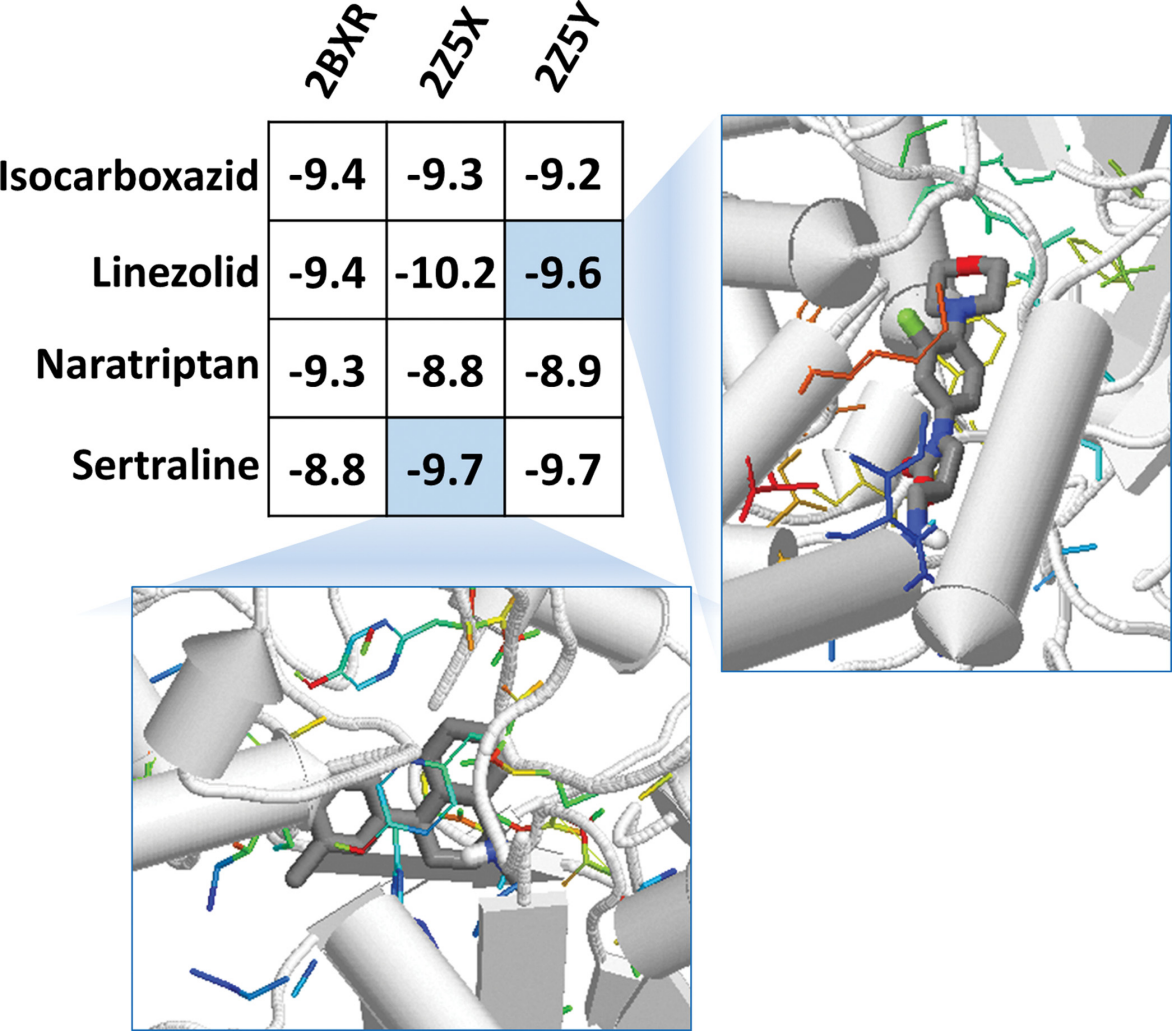
Visualization of the partial CPI for sertraline and the drug that may have interaction with it. All four drugs ranked the MAO protein structures (2BXR, 2Z5X and 2Z5Y) to the top 20% among all library proteins in their score vectors with the docking scores provided in the figure. Two 3D visualizations shown here for the two cells in CPI matrix were captured from our server.

We submitted the drug sertraline to DDI-CPI for a prediction test. The server predicts that sertraline might interact with isocarboxazid, linezolid and naratriptan. Sertraline is a selective serotonin-reuptake inhibitor for antidepressant treatment as well as a substrate of flavin-containing amine oxidase A (MAO-A) (40,41). By investigating the CPI profiles of these predicted DDIs, all of the predicted drugs can rank the MAO-A targets to the top 20% among all library proteins in their score vectors (Figure 3), indicating that these DDIs may be through MAO-A. In fact, literature reported that the three drugs predicted indeed interact with MAO-A and the interactions between sertraline and other three drugs do exist (42–44). This case study demonstrates that our server not only predicts DDIs, but also helps uncover part of the mechanisms behind-the-scene by showing the PK/PD proteins that may be involved in the interaction.

## DISCUSSION

Competition between metabolizing enzyme, transporter, or unexpected off-targets can cause DDIs (7–10,45). Fortunately, *DDI-CPI* server is the first one to provide real-time DDI predictions based only on the interactome of drugs toward a representative collection of PK/PD proteins. It serves as a complementary tool in addition to current methods that offers DDIs suggestions, and could help provide the potential mechanism explanations for any molecules with a given structure. However, as stated before, the DDIs may result from alternative mechanisms other than drug–protein interactions such as pharmaceutical interactions and drug metabolites (46). To ensure the speed of high-throughput calculations, we treated the protein targets as simplified rigid models which are not fully realistic representations (47). This assumption, although justified, could still lead to inaccurate predictions. We are not able to guarantee the docking accuracy of the user's molecule toward all proteins. Therefore, we recommend users to make the judgment based on the docking scores, the docked positions, and could even validate the binding in a more sophisticated docking or wet labs. Nonetheless, we believe the impact of false docked ligand–protein complexes could be minimized in our DDI predictions. (i) The false positives exist in both positive and negative set, thus the noise can be neutralized during our model training process. (ii) Instead of focusing on single drug–protein interaction, CPI considers the docking score vector of the drug toward all available proteins for decision making, minimizing the impact of outliers.

To evaluate whether the model performance is impacted by structural similarity of the drugs within a pair, we calculated all pairwise Tanimoto coefficients among the 2515 drug molecules in library and excluded those which have Tanimoto coefficient >0.75 toward any other molecule. We performed a 10-fold cross-validation on the new training set which contains 1620 positives and 1893 negatives. The model obtained an AUROC of 0.870 ± 0.002 and AUPR of 0.860 ± 0.003 (accuracy: 0.815 ± 0.003, precision: 0.756 ± 0.014, sensitivity: 0.861 ± 0.012, specificity: 0.780 ± 0.016 and *R*^2^: 0.410 ± 0.006), which indicates our model is less likely to be impacted by the structural similarity of the drugs within a pair.

A fundamental difference between *DDI-CPI* and *DRAR-CPI* (26) is that the object in *DDI-CPI* is drug-pair instead of single drug in *DRAR-CPI.* While *DRAR-CPI* calculates the similarity between drugs, *DDI-CPI* further utilizes the statistical model to predict the probability of a drug pair being the true DDI pair. The feature in *DRAR-CPI* is the docking score of the drug with each protein, while the novel definition for feature in this server is the combination of docking scores between each drug pairs across the entire protein set.

## CONCLUSION

- *DDI-CPI* server can predict DDI potentials between the user's drug across 2515 drug molecules in library (85% are FDA approved drugs), which is supported by the prediction results from cross-validations, independent validations and case studies.
- *DDI-CPI* can suggest putative PK/PD proteins involved in the predicted DDIs, thus could help decipher unknown mechanisms of DDI mediated by unexpected drug–human protein interactions.

## SUPPLEMENTARY DATA

Supplementary Data are available at NAR Online.

Supplementary Data
