# Supplementary material for: DDI-CPI, a server that predicts drug–drug interactions through implementing the chemical–protein interactome
Source: Nucleic Acids Res. 2014 May 29;42(Web Server issue):W46–52. doi: 10.1093/nar/gku433 (PMC4086096; doi:10.1093/nar/gku433)
Supplement: Supplementary Data [file supp_42_W1_W46__index.html]

Supplementary Data 

# *DDI-CPI*, a server that predicts drug–drug interactions through implementing the chemical–protein interactome

## Supplementary Data

**Files in this Data Supplement:**

- Supplemental Table 1
- Supplemental Table 2
- Supplemental Table 3
